# Movement Disorders Induced by SARS-CoV-2 Infection: Protocol for a Scoping Review

**DOI:** 10.3390/jcm11040923

**Published:** 2022-02-10

**Authors:** Elena Cecilia Rosca, Zsolt Vastag, Onanong Phokaewvarangkul, Jirada Sringean

**Affiliations:** 1 Department of Neurology, Victor Babes University of Medicine and Pharmacy of Timisoara, Eftimie Murgu Sq. No. 2, 300041 Timisoara, Romania; 2 Department of Neurology, Clinical Emergency County Hospital Timisoara, Bd. Iosif Bulbuca No. 10, 300736 Timisoara, Romania; 3 Doctoral School, Victor Babes University of Medicine and Pharmacy of Timisoara, Eftimie Murgu Sq. No. 2, 300041 Timisoara, Romania; vastag_zsolty@yahoo.com; 4 Clinical Hospital of Infectious Diseases and Pneumology Victor Babes Timisoara, Gh. Adam Street No. 13, 300173 Timisoara, Romania; 5 Chulalongkorn Center of Excellence for Parkinson’s Diseases and Related Movement Disorders, Department of Medicine, Faculty of Medicine, Chulalongkorn University, Bangkok 10330, Thailand; oji@chulapd.org (O.P.); jsr@chulapd.org (J.S.)

**Keywords:** movement disorders, SARS-CoV-2, COVID-19, systematic review

## Abstract

Infections are a significant cause of movement disorders. The clinical manifestations of SARS-CoV-2 infection are variable, with up to one-third of patients developing neurologic complications, including movement disorders. This scoping review will lay out a comprehensive understanding of movement disorders induced by SARS-CoV-2 infection. We aim to investigate the epidemiology, clinical and paraclinical features, interventions, and diagnostic challenges in patients with different types of movement disorders in the context of SARS-CoV-2 infection. We will search three databases applying appropriate search terms. Inclusion and exclusion criteria are pre-defined; the data of eligible studies will be extracted in standardized forms. We will report the results following Preferred Reporting Items for Systematic reviews and Meta-Analyses extension for Scoping Reviews (PRISMA-ScR). We will present information for clinicians and other healthcare professionals, policymakers, and public health researchers. In addition, the results of the present review may assist in the development and confirmation of inclusion criteria and research questions for further systematic review or meta-analysis, with more precise, narrower questions.

## 1. Introduction

Movement disorders are neurologic syndromes with either an excess or a paucity of movement, unrelated to weakness or spasticity. Among the hyperkinetic movement disorders, the most frequent are tremor, dystonia, myoclonus, chorea and athetosis, ballism, tics, and sleep-related movement disorders. Hypokinetic movement disorders include parkinsonism and rigidity.

Infections are a significant cause of movement disorders, as up to 20% of movement disorders are due to an infectious cause [1]. The most frequent agents are beta-hemolytic group A streptococcus, the flavivirus causing Japanese encephalitis, HIV, West Nile virus, and Creutzfeldt–Jakob disease [2].

Two mechanisms were postulated to underly the development of infection-related movement disorders. First, they can be a direct consequence of an active infection in relevant cerebral structures; second, they can be a manifestation of a delayed immune-mediated process secondary to previous infection [3]. In addition, the role of neuroinflammation in neurodegeneration has started to attract interest in recent years. A possible link between neuroinflammation and parkinsonian syndromes such as encephalitis lethargica and postencephalitic parkinsonism [4] was investigated in the context of Spanish flu. Nonetheless, the subject is still under debate, as there is no proven correlation yet. The role of the viral stimulation of microglial activation in neurodegeneration has regained attention in the context of the SARS-CoV-2 infection. Nonetheless, there is a lack of long-term observations, and the question of whether there is any correlation between SARS-CoV-2 and morbidity in parkinsonian syndromes remains open. Meanwhile, recent studies highlighted the impact of the virus on the central nervous system, demonstrating a fast viral spread in the regions connected with the olfactory bulb, including the basal ganglia, with increased neuronal death. These findings indicate the potential for long-term consequences of coronavirus disease 2019 (COVID-19) [5].

Therefore, infection-related movement disorders may have an acute or subacute onset or can be delayed months to years after the infection. However, most movement disorders present about six weeks from the onset of infection (but depends on the cause) [6,7].

Regarding the treatment, a multifaceted approach is frequently used to control the patient’s symptoms. The majority of infection-related movement disorders are a direct consequence of an active infectious process that affects the brain structures implied in the motor network [8]. Therefore, the main treatment consists of disease-specific, infection-targeted medication. In other cases, the movement disorders are caused by a delayed immune-mediated process triggered by a previous infection and may respond to immunomodulatory treatments [7,8,9]. In addition, symptomatic treatment may be used [7,8,9].

The SARS-CoV-2 virus is a novel agent spreading rapidly. The clinical manifestations of COVID-19 are diverse, from asymptomatic to severe disease. Furthermore, up to one-third of the patients with SARS-CoV-2 infection develop neurologic complications, including movement disorders [3]. The most frequent movement disorders were reported to be myoclonus and ataxia [10,11], but patients may also present with chorea, tremor, or dystonia [11]. Interestingly, previous reviews reported a predominance of hyperkinetic movement disorders, while hypokinetic disorders were rare [12]. However, due to the novelty of SARS-CoV-2 infection, the management of patients is based mainly on other respiratory infections, mainly those caused by influenza and other coronaviruses.

The objective of the present scoping review is to provide a thorough insight into the existing literature by reporting data on movement disorders induced by SARS-CoV-2 infection. We aim to investigate the epidemiology, clinical and paraclinical characteristics, and the diagnostic and therapeutic challenges in patients with different types of movement disorders in the context of SARS-CoV-2 infection. Compared to classic systematic reviews that address relatively specific, focused questions, a scoping review will provide a broader perspective on this subject. It is used to map and investigate the emerging scientific evidence when it is not clear yet what other, more precise research questions can be asked and usefully addressed. Consequently, a scoping review is the most suitable method to assess the current scientific knowledge on these neurological aspects of COVID-19.

In addition, we aim to report significant policy implications, highlighting research gaps that require attention, and provide recommendations on SARS-CoV-2 induced movement disorders reporting.

## 2. Materials and Methods

In evidence-based medicine, there are over 20 types of systematic reviews [13,14]. Considering our questions and objectives, we determined that the most appropriate synthesis method to be used in the present case is a scoping review. In addition, we used an online tool designed to aid researchers in deciding which knowledge synthesis method would be most suitable for their particular research questions (https://whatreviewisrightforyou.knowledgetranslation.net/, accessed on 9 December 2021) [15,16].

Furthermore, as the myoclonus is the most frequent movement disorder induced by SARS-CoV-2 infection, we performed a brief literature search to understand the extent of the research that exists on this topic. Our scoping search investigated how many systematic reviews are indexed in LitCOVID and the WHO database on COVID-19 (to 23 January 2022). We used the terms “myoclonus” AND “review” and found 28 results. After deduplication, 18 articles were included. Abstract screening further reduced the number of included papers to 10. Three narrative reviews were excluded from the ten articles assessed in full text as the authors did not perform a systematic literature search. Seven reviews used a systematic search [10,11,17,18,19,20,21]. However, two reviews searched only one database [11,19], limiting the number of possible published cases. In addition, another review searched PubMed and Medline, which is a subset of PubMed [17]. A narrative review searched PubMed and a preprint server [10]. The date of three published reviews is relatively old for the rapidly evolving research on COVID-19 [11,17,20], and several authors did not specify the date of their searches [18,21]. The main characteristics of the reviews on myoclonus in the context of SARS-CoV-2 infection are presented in Table 1.

Therefore, a scoping review on the movement disorders associated with SARS-CoV-2 infection is timely and could provide valuable insights into the spectrum of these neurological disorders.

### 2.1. Research Questions

To identify important issues that need to be addressed, we took into account several aspects of movement disorders induced by SARS-Co-V-2 infection. Accordingly, we developed research questions that clearly defined the population, concept, and context (PCC) of the systematic review [22].

For the present scoping review, we identified several research questions:

- Does SARS-CoV-2 infection cause movement disorders?
- If yes, what types of movement disorders are present in COVID-19 patients?
- What was the severity of COVID-19?
- Did the patient have previous movement disorders?
- What are the published data on the epidemiology of movement disorders induced by SARS-CoV-2 infection?
- What is the timing of the movement disorder after the SARS-CoV-2 infection (i.e., acute, subacute, or chronic)?
- Which are the clinical features?
- What do we know about the diagnostic tools?
- What interventions might work?
- Which are the presumptive mechanisms underlying movement disorders?

### 2.2. Search Strategy and Eligible Studies

In order to develop our search strategy, we followed the recommendations for scoping reviews by the Joanna Briggs Institute [22] and used the PCC mnemonic.

A bibliographic search will be performed on the following academic research databases: World Health Organization COVID-19 Database (https://search.bvsalud.org/global-literature-on-novel-coronavirus-2019-ncov/, accessed on 9 December 2021), LitCOVID (https://www.ncbi.nlm.nih.gov/research/coronavirus/, accessed on 9 December 2021) and a preprint website, namely MedRxiv (https://www.medrxiv.org/, accessed on 9 December 2021). In addition, we will review reference lists of all relevant articles and the MDS website (case repository) to identify further possible studies.

As the aforementioned databases contain curated literature for SARS-CoV-2 infection, we will use only the following keywords: “parkinsonism”, “parkinson”, “ataxia”, “myoclonus”, “tremor”, “chorea”, “dystonia”, “hypokinesia”, “hyperkinesia”, and “tics”. Our objective is to produce a comprehensive list of studies suitable for inclusion in our review; therefore, we will not apply any search filters. Additionally, we will not apply any language restrictions. If the primary selected studies or reviews are missing important information, we may contact the authors for further data.

### 2.3. Selection of Studies

Following the PCC mnemonics, our scoping review will include research reporting data on adults (over 18 years old) and children (P), investigating patients with movement disorders (C) in the context of infection with SARS-CoV-2 (C).

We will not set any limits on publication date, setting, or study design. Additionally, we will not exclude cases with pre-existing movement disorders that developed new movement disorders in the context of COVID-19. The pre-existing movement disorders will be noted among the patient’s comorbidities.

We will include prospective and retrospective observational/interventional studies, including:

− Case reports or case series;
− Interventional studies (randomized trials and clinical reports);
− Outbreak reports;
− Case–control studies;
− Studies that incorporate models to present observed data, but we will exclude studies reporting only predictive modeling.

Nonetheless, we plan to include only quantitative studies; we will exclude qualitative studies since we do not plan to investigate barriers or facilitators for interventions. If relevant and available, we will include primary research and systematic reviews.

For the screening process, we will use a two-stage approach. In the first stage, two reviewers will independently evaluate the eligibility of the study based on the inclusion and exclusion criteria by reviewing the titles and abstracts of all identified articles. We will assess the full text of any article considered eligible by either or both reviewers. In the next stage, the text of the selected articles will be independently screened by two authors, and a third author will solve any disagreements.

### 2.4. Data Extraction

In order to provide a descriptive summary of the findings, we will chart the results. At the protocol stage, we developed a draft charting table to collect essential information of the source, including data on authors, publication year, location and setting of the study, research methods, and results or findings that are relevant to our review questions.

We will extract the following patient data: age, gender, immunological status (vaccination), comorbidities, onset, clinical data (general clinical signs, neurological examination), laboratory investigations (general, SARS-CoV-2 infection, CSF analysis), imaging (including pulmonary CT/MRI, and neuroimaging), pathological samples (biopsy/autopsy), treatment (general, neurological), evolution and assumed mechanism of neurological impairments. If applicable, depending on the type of the study, we will extract other additional research data.

For studies reporting interventions, we will group the information into three subgroups: pharmacological, nonpharmacological, and a combination of pharmacological and nonpharmacological interventions. In addition, we will extract data on the comparator (if available) and other details such as the duration of the treatment and outcomes.

We may further refine and update the charts if it emerges that additional unanticipated data can be usefully tabulated. However, we will develop piloted data extraction forms on the first three studies from each category of movement disorders to ensure all important and relevant information is extracted.

The data will be extracted into the final tables by two independent reviewers; any discrepancies will be solved by a third author.

### 2.5. Reporting the Findings and Summarizing the Results

The findings of the review will be presented in accordance with Preferred Reporting Items for Systematic reviews and Meta-Analyses extension for Scoping Reviews (PRISMA-ScR) recommendations [23,24].

We will present the characteristics of the included studies, providing a descriptive numerical summary. We will classify the results under the main conceptual categories, including “parkinsonism”, “ataxia”, “myoclonus”, “chorea”, “dystonia”, “tremor”, and “tics”. Under this grouping, we will detail further the data on the characteristics of the studies, including the total number of patients, types of study design, date of publication, and main findings.

In addition, to ensure an organized presentation of the results, we will use tables and figures in agreement with the objectives and the scope of the review. Additionally, we will chart the results as distribution of research by type of movement disorders and vaccination status (if applicable).

Finally, the findings will be elaborated, considering the research and practice to support us in identifying gaps in this research area.

Towards the end of the review, when we will have considerable awareness of the published data, we will further refine the presentation of the results.

### 2.6. Assessment of Quality of the Included Studies

The scope of the present review is to map the information that has been published in the area of movement disorders induced by SARS-CoV-2 infection. Consequently, we will not formally assess the methodological quality of the included research. The main distinction between scoping reviews and other types of systematic reviews is that scoping reviews allow a synopsis of the current evidence, disregarding the risk of bias of the included studies [22].

## 3. Discussion

Although several reviews on movement disorders associated with SARS-CoV-2 infection have been published, there are important reasons that warrant us to perform a new systematic review. First, most of the reviews were performed several months ago, and the rapidly evolving knowledge in the field requires new updates. Second, several authors searched for relevant studies only in the PubMed database. Recent research estimates that 60% of the reviews using only PubMed are likely to miss more than 5% of references that are relevant to the subject [25]. Authors suggest that an optimal search should include Embase, MEDLINE, Web of Science, and Google Scholar as a minimum requirement to warrant efficient and adequate coverage [25]. Therefore, we decided to use two databases that will allow us an efficient search. The first database, LitCOVID, is a curated literature database providing access to 214755 (and growing) relevant articles in PubMed. The second database, provided by WHO, represents a comprehensive multilingual source of current literature on the SARS-CoV-2 infection, indexing articles from Embase, Scopus, Web of Science, ProQuest Central, grey literature, and several other databases. In addition, we intend to search MedRxiv, a preprint database.

Our findings may be limited by the quality and breadth of the data in the case reports, which may not be uniform or consistent in all papers. However, case reports represent a relevant, appropriate, and essential study design in promoting scientific knowledge, especially in the case of rare disorders. Although the methodological limitations of case studies in the analysis of treatments and the development of new tests are well known, observing single patients can provide useful insights on the etiology, pathogenesis, natural history, and treatment, especially in rare disorders [26].

Furthermore, case reports and case series have significantly influenced medical knowledge and continue to promoter scientific research and understanding [27]. Although several concerns were raised about the high likelihood of bias associated with single case reports or case series and the weak inferences they may provide, such observations are an important basis for learning by pattern recognition and further progress of medical knowledge [27]. For example, a systematic review of the cases with lipodystrophy enabled authors to propose the core and supportive clinical features of the disease and to narratively present the data on available treatment options [28]. Additionally, another systematic review, including 172 cases of glycogenic hepatopathy, a rare disorder, warranted for the first time the characterization of the patterns of liver enzymes and hepatic injury in these patients [29].

In addition, compared to cohort studies, case reports contain much more data on an individual patient [30]. However, we will consider any type of study for inclusion, providing it reports sufficient detail and relevant information.

A scoping review is, in the case of SARS-CoV-2-induced movement disorders, the most pertinent approach that allows for a comprehensive investigation of the research questions. Hence, a descriptive mapping of the published research in this area is the most suitable review method. The scoping review will warrant us to respond to our research questions with clear, up-to-date answers. In addition, we will identify any research gaps in this area, providing details on critical implications for research and any requirement for further primary research or systematic reviews [15,22].

The key findings will be detailed, allowing their use to inform clinical practice. However, implications for practice may be hampered because of the lack of the methodological quality assessment of included studies [22]. In addition, practice recommendations will not be graded because we will not provide a rating of quality or levels of evidence.

We will discuss the results in the context of the current, up-to-date, international research, practice, and policies. In addition, we will present any potential limitations of the studies included in the review.

In the end, we will provide a general, overall conclusion according to our objectives and questions.

## 4. Conclusions

To date, several reviews on different movement disorders in COVID-19 patients have been performed. However, to the best of our knowledge, most searches are up to December 2020, and the reviews report only disparate aspects of movement disorders in SARS-CoV-2 infections. There has been no previous systematic review aiming to present such a detailed picture of patients with this pathology, covering all the critical aspects of the disease. Consequently, the outcomes of our evidence-based approach will provide significant information to clinicians and other healthcare professionals, policymakers, and public health scientists. In addition, the results of the present scoping review may provide a basis to inform further systematic review or meta-analysis, with narrower, more focused questions, assisting in the development and confirmation of inclusion criteria and further research questions. Publication of the present protocol supports an explicit and transparent methodology for our scoping review, avoiding possible problems we may encounter while performing the review.

## Figures and Tables

**Table 1:** Characteristics of the reviews on myoclonus in the context of SARS-CoV-2 infection.

Article	Databases	Date of Search	Findings	Notes
Brandao 2021 [11]	PubMed	Up to 25 January 2021	59/93 cases presented myoclonus	Investigated movement disorders among patients with SARS-CoV-2 infection.
Chan 2021 [17]	PubMed and Medline	Up to 6 December 2020	51 cases of myoclonus or ataxia	Investigated myoclonus and cerebellar ataxia associated with SARS-CoV-2 infection.
Giannantoni 2021 [18]	PubMed and Cochrane Library	The date is not specified	6 cases	Investigated myoclonus and ataxia in COVID-19 patients.
Hirschfeld 2021 [19]	PubMed	Up to 31 July 2021	33 cases of myoclonus	Among the autoimmune- mediated hyperkinetic movement disorders, the most common was ataxia (83.67%), followed by myoclonus (67.35%).
Roy 2021 [20]	PubMed and Google Scholar	Up to 30 May 2020	4 cases with myoclonus	The main focus of the paper was on the neurological and neuropsychiatric impacts of the COVID-19 pandemic.
Salari 2021 [21]	PubMed and Scopus	The date of the search is not specified	64 patients with movement disorders	Limited data on myoclonus.
Schneider 2021 [10]	PubMed and MedRxiv	Up to August 2021	More than 50 cases	The exact number of myoclonus cases is not specified. Narrative review.

## Data Availability

This systematic review will use only data published in scientific journals or on preprint websites and thus in the public domain.
